# Cannabis dabbing: An emerging trend

**DOI:** 10.1097/01.NURSE.0000743108.72528.d8

**Published:** 2021-04-23

**Authors:** Mary Frances Mullins

**Affiliations:** **Mary Frances Mullins** is a clinical nurse educator at Memorial Medical Center in Modesto, Calif., and holds a graduate certificate in Contemporary Theory in Addictive Behavior.

**Keywords:** BHO, butane hash oil, cannabis, cannabis concentrates, cannabis dabbing, cannabis withdrawal syndrome, CWS, tetrahydrocannabinol, THC

## Abstract

Cannabis dabbing refers to the recreational inhalation of extremely concentrated tetrahydrocannabinol, the main psychotropic cannabinoid derived from the marijuana plant. The practice carries significant health and legal risks. This article discusses what nurses need to know about dabbing and how they can educate patients who may be engaging in risky behavior.

## Abstract 2

Cannabis dabbing is the recreational use of extremely concentrated tetrahydrocannabinol, the main psychotropic cannabinoid derived from the marijuana plant. In this article, the author details the significant health and legal risks associated with dabbing and discusses how nurses can educate patients.

**Figure:**
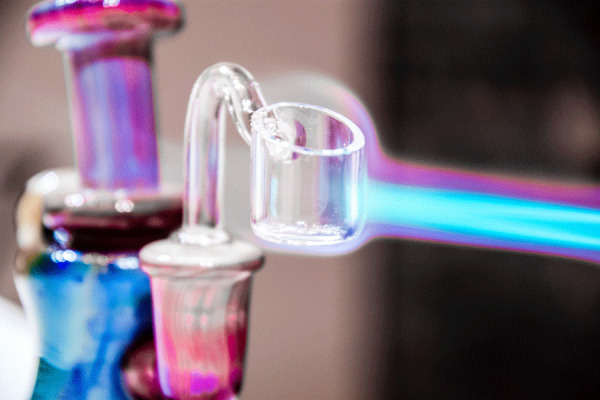
No caption available.

CANNABIS DABBING refers to the use of concentrated marijuana, a practice that has significant health risks.^1^ According to the CDC, cannabis dabbing is increasingly popular among teenagers and young adults in the US.^2^,^3^ The legalization of recreational and medical cannabis in many states has corresponded with a flourishing cannabis merchandise industry and a rise in new types of cannabis use, including cannabis dabbing.^4^

As patient educators and advocates, nurses are in a prime position to educate patients and the public about the potential health hazards and legal risks of cannabis dabbing. This article discusses what nurses need to know and how they can educate patients who may be engaging in risky behavior.

## What is cannabis dabbing?

The term *dabbing* is jargon that refers to the inhalation of vapors derived from marijuana-based oils, concentrates, and extracts.^2^,^5^ Historically, marijuana has been consumed by smoking (joints, blunts, pipes), inhalation through a bong device, and oral consumption (edibles). Cannabis dabbing involves inhaling waxy cannabis preparations consisting of extremely concentrated tetrahydrocannabinol (THC), the main psychotropic cannabinoid derived from the marijuana plant, through a vaporizer or similar device.^1^ THC is the ingredient responsible for the “high” associated with marijuana use—feelings of euphoria, increased insightfulness, heightened sociability, sexual pleasure, and relaxation that many users say that they experience and enjoy.^6^-^8^

Cannabis concentrates can be prepared in a home using amateur equipment or in a commercial lab.^9^ The various ways in which cannabis concentrates can be produced include water-based processing, dry processing, dry ice processing, and solvent-based processing. Solvent-based processing includes the use of carbon dioxide, a nonflammable solvent, and flammable solvents such as propane, alcohol, ether, or butane (lighter fluid).^7^,^10^

A popular noncommercial method for preparing cannabis concentrates is to use butane oil as a solvent to extract THC from dried marijuana. The resulting product, butane hash oil (BHO), has stronger effects than conventional flower cannabis.^1^ Other names for BHO are amber, glass, black glass, wax, ear wax, shatter, dabs, liquid THC, budder, butter, honeycomb, and butane honey oil depending on the consistency of the product.^3^,^7^,^9^,^11^,^12^

## Making BHO at home

Home manufacturing of BHO, known colloquially as *blasting*, is becoming increasingly prevalent because the production process is uncomplicated and requires few resources.^13^ Numerous instructional videos are available on internet and social media sites. Using butane as the solvent is commonplace because it is relatively inexpensive and produces high THC levels with enduring effects.^8^,^13^

The process entails passing butane oil through a glass or steel tube loaded with dried cannabis trimmings. THC and other hydrophobic compounds within the dried cannabis dissolve into the butane; the THC-butane solution then exits the tube through a filtering device.

To prepare the cannabis concentrate for inhalation, a small amount, or *dab*, of the crystalized resin is placed on a shallow tray or dish, called a *nail,* attached to a waterpipe that has been preheated with a blowtorch.^13^ Some nails include an additional piece called a *dome*, which helps evenly distribute heat and retain vapor.^14^ Due to its volatile nature, the butane evaporates, leaving behind crystalized resins.

Solvent-based products have an average THC concentration of 54% to 69% but may reach 80% or more. In contrast, the concentration of THC in conventional cannabis is only 10% to 15%.^7^,^15^

The high THC concentration in BHO dabs makes health risks of using BHO much more serious than using marijuana in traditional ways (see *How THC affects the endocannabinoid system*). In addition, these high concentrations are delivered to users at once in a single breath, increasing the risk of physical dependence and addiction. Higher doses of THC are also more likely to produce anxiety, agitation, paranoia, and psychosis.^7^ The following discussion focuses on these and other specific dangers related to the recreational use of cannabis concentrates.

## Fire hazards

A significant threat associated with BHO dabbing is the risk of fire or explosion during the production of homemade BHO. Production of concentrated cannabis extracts by licensed commercial manufacturers typically does not carry the same risk of detonation because these facilities use a closed-loop system that recycles the solvent, unlike noncommercial open systems that allow butane gas to escape.^9^

Handcrafted BHO dabs are typically formed by blasting butane gas from a torch through the dried marijuana plant material.^15^ Butane is an unstable, highly combustible compound. Butane gas can accumulate in confined spaces, such as garages, mobile homes, and vacant houses—common places for BHO manufacture used by nonprofessional entrepreneurs—and is easily ignited by any flame source, including the spark produced by static electricity.^16^ Many explosions have occurred during the recreational production of BHO, some causing death or serious burn injuries requiring skin grafting.^7^,^13^,^15^

## Legal risks

Besides being dangerous, the manufacture of BHO is a violation of US federal law, Title 21, US Code, Section 841.^17^ Illegal operations are called honey oil labs.^18^ According to Gordon, the legal penalties (as well as the risk of explosion) associated with the home production of BHO are similar to the risks of operating a methamphetamine lab.^15^ Even in states where recreational marijuana has been legalized, such as California and Colorado, the production of hash oil using butane or other flammable liquids is strictly prohibited.^7^

Heard and colleagues describe the destructive results of a home BHO lab that exploded, leaving a 20-year-old man with second-degree burns to his torso, face, and hands. Upon his recovery, he was arrested for manufacturing BHO.^19^

This is an important consideration for those who manufacture BHO, whether for personal use or for sale. Prison sentences have been given to BHO producers following drug lab explosions.^17^

## Contaminants

Homemade dabs and/or dabs obtained from an undependable source may contain residual solvents and other toxic substances including butane, pesticides, and conceivably carcinogenic contaminants.^20^ Microbial pathogens, particularly fungi and bacteria, may also be present in cannabis preparations, often because of improper preparation and storage.^21^ Depending on the substance, these impurities may pose immediate or delayed health risks of their own. Future research may illuminate specific health consequences related to dab contaminants.

## Physiologic and psychological effects

The use of BHO has been linked to lung injuries secondary to the inhalation of concentrated cannabis. Case reports in the literature describe BHO dabbing-induced acute and chronic lung impairment, including pneumonia, pneumonitis, and acute hypoxemic respiratory failure.^22^ Dabbing of BHO is also associated with the development of structural brain changes, psychosis, and cognitive dysfunction. Cases of emergent psychosis following use of cannabis dabs have been reported.^23^,^24^

Other physiologic and psychological disorders associated with the use of BHO include tachycardia, hypertension, antegrade amnesia, temporary loss of consciousness, lethargy, visual/auditory hallucinations, and tactile and visceral hallucinations.^15^,^20^ End-organ damage through serotonergic and sympathomimetic neural pathways has also been reported.^25^

High doses of THC delivered when BHO is inhaled can result in agitation and feelings of intense anxiety.^26^ Repetitive consumption of concentrated cannabis may lead to short-term attention deficits, memory impairment, and mood alterations. Pierre recommends that nurses query their patients about the potency of the marijuana products that they use to accurately assess the risk of adverse psychiatric reactions.^24^

Freeman and Winstock report that the higher concentrations of THC in BHO compared with nonconcentrated cannabis composites are associated with an increased risk of addiction and physical dependence.^26^ According to Bonnet and Preuss, cannabis addiction and dependence result in withdrawal symptoms upon cessation of use in up to 90% of cases and is associated with heavy, frequent, or prolonged cannabis use.^27^

Nurses must be knowledgeable about signs and symptoms of cannabis withdrawal syndrome (CWS), which is likely to follow the discontinuation of prolonged and/or heavy use of cannabis. The most common signs and symptoms of CWS are nervousness, anxiety, irritability, depression, insomnia, anorexia, tremors, diaphoresis, headache, chills, fever, and abdominal pain.^27^-^29^

Little detailed, informative literature for professionals and researchers regarding treatment for addiction to BHO dabbing exists, hindering an optimal response to the issue.^9^ Many substance abuse rehabilitation centers in the US maintain that they provide BHO dabbing rehabilitation services. These facilities assert that rehabilitation for BHO dabbing is no different than rehabilitation for other marijuana use. Behavioral modification treatments including contingency management (which involves using incentives or rewards to reinforce positive behaviors), cognitive behavioral therapy, and motivational interviewing are listed on many of rehabilitation facility websites. Research indicates that incentive-based interventions can be effective in promoting treatment retention and abstinence from drugs.^30^,^31^

## Education and research

Because nurses are highly regarded as experts and educators by the public, they are well positioned to provide patients with accurate information, advice, and support to promote health and well-being. As an emerging trend, the far-reaching impacts of BHO dabbing are still unclear and the full extent of long-term detrimental health effects are not yet known. More research is needed. To this end, nurse researchers and scholars may be in an ideal position to bring valuable data regarding the phenomenon of BHO dabbing to light.

## How THC affects the endocannabinoid system^7^,^8^

The chemical structure of THC is similar to anandamide (an endogenous cannabinoid), which accounts for the body's ability to recognize it and for its ability to alter natural brain communication. Endogenous neurotransmitters such as anandamide send chemical signals between neurons throughout the nervous system, affecting areas in the brain that influence memory, pleasure, concentration, thinking, coordination, movement, and time and sensory perception.

Due to its similarity to endogenous cannabinoids, THC can attach to cannabinoid receptors in the brain and disrupt normal neurotransmission, resulting in alterations to these and other neurologic functions. Because THC disrupts neurotransmission in the basal ganglia and cerebellum—areas of the brain that regulate balance—reaction times, posture, and dexterity may temporarily be compromised.

THC can also affect other regions of the brain including the hippocampus and orbitofrontal cortex, which control attentional focus and the ability to form memories. Consequently, cannabinoid compounds can impair thought processes and interfere with an individual's capability to learn and perform complex tasks.

Acting on cannabinoid receptors in the nucleus accumbens and other brain areas, THC activates the reward system by stimulating neurons to release high levels of the neurotransmitter dopamine. The subsequent flood of dopamine contributes to the euphoric high that many cannabis users seek.
